# Is Gamma Radiation Suitable to Preserve Phenolic Compounds and to Decontaminate Mycotoxins in Aromatic Plants? A Case-Study with *Aloysia citrodora* Paláu

**DOI:** 10.3390/molecules22030347

**Published:** 2017-02-23

**Authors:** Eliana Pereira, Lillian Barros, Amilcar L. Antonio, Sandra Cabo Verde, Celestino Santos-Buelga, Isabel C. F. R. Ferreira, Paula Rodrigues

**Affiliations:** 1 Mountain Research Centre (CIMO), ESA, Polytechnic Institute of Bragança, Campus de Santa Apolónia, 1172, 5300-253 Bragança, Portugal; eliana@ipb.pt (E.P.); lillian@ipb.pt (L.B.); amilcar@ipb.pt (A.L.A.); 2 Grupo de Investigación en Polifenoles (GIP-USAL), Facultad de Farmacia, Universidad de Salamanca, Campus Miguel de Unamuno s/n, 37007 Salamanca, España; csb@usal.es; 3 Laboratory of Separation and Reaction Engineering (LSRE), Associate Laboratory LSRE/LCM, Polytechnic Institute of Bragança, Campus de Santa Apolónia, 1134, 5301-857 Bragança, Portugal; 4 Centro de Ciências e Tecnologias Nucleares (C2TN), IST, Universidade de Lisboa, Estrada Nacional 10 (km 139.7), 2695-066 Bobadela LRS, Portugal; sandracv@ctn.tecnico.ulisboa.pt

**Keywords:** gamma radiation, herbs, phenolic compounds, aflatoxin B1, ochratoxin A, chromatography

## Abstract

This study aimed to determine the effect of gamma radiation on the preservation of phenolic compounds and on decontamination of dry herbs in terms of ochratoxin A (OTA) and aflatoxin B1 (AFB1), using *Aloysia citrodora* Paláu as a case study. For this purpose, artificially contaminated dry leaves were submitted to gamma radiation at different doses (1, 5, and 10 kGy; at dose rate of 1.7 kGy/h). Phenolic compounds were analysed by HPLC-DAD-ESI/MS and mycotoxin levels were determined by HPLC-fluorescence. Eleven phenolic compounds were identified in the samples and despite the apparent degradation of some compounds (namely verbasoside), 1 and 10 kGy doses point to a preservation of the majority of the compounds. The mean mycotoxin reduction varied between 5.3% and 9.6% for OTA and from 4.9% to 5.2% for AFB1. It was not observed a significant effect of the irradiation treatments on mycotoxin levels, and a slight degradation of the phenolic compounds in the irradiated samples was observed.

## 1. Introduction

There is currently a high demand for medicinal and aromatic plants, due to their combined aromatic and bioactive properties [1,2]. One of the most significant compounds which confer bioactive potential to plants are the phenolic compounds. These have been largely studied based on their therapeutic properties related to the prevention of chronic inflammation, cardiovascular problems, cancer, and diabetes [2]. Some studies claim that the absorption of these compounds in the body, occur in different routes linked to the gastrointestinal tract, where microorganisms, enzymes, and even glucose transporters are involved. The partial release of polyphenols occurs in the gastrointestinal lumen, where they are metabolized and rendered absorbable, so that they can exert their health benefits [3]. Nevertheless, the use of these medicinal and aromatic plants do not always adhere to the industrial and commercial tough requirements of quality and safety. Natural contamination of plant material by fungi and associated toxins during growth, harvesting, storage and drying processes, in general, presents a threat to public health [4]. Medicinal and aromatic plants are no exception, as they are frequently contaminated with numerous toxigenic fungi.

There are more than 400 compounds classified as mycotoxins and, among them, aflatoxins (AF), and ochratoxin A (OTA) are the best studied. AF are produced by *Aspergillus flavus* and some closely related species. AFB1 is the most common aflatoxin contaminating food products; it is reported as the most toxic and carcinogenic compound naturally produced, being classified as Group 1 carcinogen [5,6]. The mutagenic and carcinogenic effects of AFB1 in various animals have been documented, and different epidemiological studies showed the existence of a correlation between human liver cancer and the levels of this mycotoxin in the diet [7,8]. OTA is produced by several *Aspergillus* and *Penicillium* species and known as a nephrotoxic, hepatotoxic, neurotoxic, teratogenic and immunotoxic agent. Its presence in the diet has been associated with a fatal human kidney disease, referred to as Balkan Endemic Nephropathy (BEN), and with an increased incidence of tumors of the upper urinary tract [9,10,11]. It is classified as Group 2B potentially carcinogen [5].

The natural occurrence of mycotoxins in plants has been frequently reported, some examples being traditional medicinal and aromatic herbs from several Asian and African countries reported to contain exceeding levels of aflatoxins and OTA [11,12,13]. Although there is no specific legislation regulating mycotoxin levels in these herbs, European regulations set maximum levels of 5 µg/kg of AFB1 and 15 µg/kg of OTA for several spices (CE No. 165/2010 and CE No. 594/2012). Various techniques have been applied in the decontamination as well as in the preservation of bioactive compounds in medicinal and aromatic plants, including irradiation [14]. This is a physical process in which plants are exposed to high-energy ionizing radiation with the aim of improving food safety and shelf life [15,16,17]. Irradiation is being increasingly recognized as a safe and efficient food processing method due to its positive effects in preservation, reduction of natural losses caused by physiological processes (budding, maturation, and aging), and elimination or reduction of microorganisms and their toxins, parasites and pests, without causing chemical changes to the food [17]. Additionally, irradiation is considered a safe process since it has not been associated with unsafe residues, as well as it reduces the dependence on chemical fumigants and preservatives traditionally used in the food industry [14,16,18]. Irradiation is currently approved by national legislations in over 55 countries worldwide [19]. European legislation [20] establishes a short list of foodstuffs authorized for irradiation treatment which includes dried aromatic herbs, spices, and vegetable seasonings, with a permitted maximum average absorbed dose of 10 kGy.

Our research group previously demonstrated that gamma radiation does not significantly change the chemical profile of dried medicinal and aromatic plants, when applied at the authorized doses [21]. Taking into account previous reports, where irradiation is described as an excellent methodology to process and decontaminate products [14], the aim of the present study was to evaluate the effect of this technology in the preservation of phenolic compounds and also on the decontamination of AFB1 and OTA in dried herbs, using *Aloysia citrodora* Paláu as a case-study.

## 2. Results and Discussion

**Figure 1:**
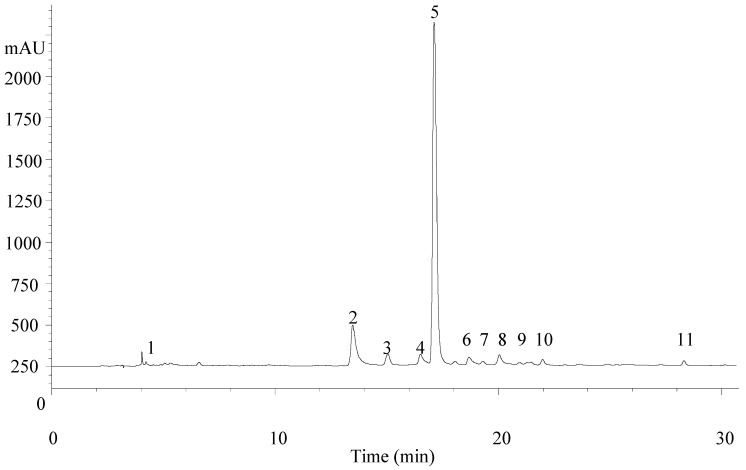
*Aloysia citrodora* phenolic profile recorded at 280 nm. Peak numbering is the same as in Table 1 and Table 2.

Data on phenolic compounds identification by HPLC-DAD-ESI/MS (High performance liquid chromatography coupled with a diode array detector and electrospray ionization tandem mass spectrometry) recorded in the negative ion mode (retention time, λ_max_ in the visible region, deprotonated molecules *m*/*z* values), the low-energy collision induced dissociation tandem mass spectrometric (CID) fragmentation pathways analysis, and tentative product ions identification are presented in Table 1. The phenolic profile of the control sample, recorded at 280 nm, is shown in Figure 1. Up to eleven phenolic compounds were detected and tentatively identified in the samples. Five of the identified compounds corresponded to caffeoyl phenylethanoid derivatives (peaks 5, 7, 8, 10, and 11), three to flavone derivatives (peaks 2, 4, and 6) and the remaining ones to a phenylethanoid glycoside (peak 1), a hydroxycinnamic acid (peak 3) and a flavonol (peak 9). Compounds were identified based on their mass and UV-VIS spectra and retention characteristics. The majority of the detected compounds (verbasoside, luteolin-7-*O*-diglucuronide, apigenin-7-*O*-diglucuronide, verbascoside, chrysoeriol-7-*O*-diglucuronide, isoverbascoside, forsythoside, eukovoside, and martinoside) have already been reported in *A. citrodora* (Table 1; [22,23,24], which has been used to support compounds identities. Compounds **3** (*p*-coumaric acid) and **9** (isorhamnetin-3-*O*-glucuronide) were identified by comparison with authentic standards. As far as we know, these two compounds have not been previously reported in *A. citrodora*.

The most abundant compound present in all samples was verbascoside (compound **5**, Table 2), a caffeoyl-phenylethanoid glycoside with antioxidant, anti-inflammatory, and antimicrobial activities, as well as wound healing and neuroprotective properties claimed to be beneficial in human health [25,26]. The effect of gamma radiation on the phenolic compounds of the studied samples presented statistically significant differences for some compounds (*p* < 0.05). The results showed a slight decrease of the major compound (verbascoside) at all the applied doses, as well as in the levels of total flavonoids (TF) and total phenolic compounds (TPC), as determined by HPLC-DAD (Table 2). However, the concentrations of verbasoside, *p*-coumaric acid, isoverbascoside, forsythoside, eukovoside, and martinoside did not change significantly (*p* > 0.05) when the maximum dose (10 kGy) was applied. The dose of 1 kGy stood out from the other applied doses, because it slightly preserved more of the phenolic compounds, in which three out of the eleven identified phenolic compounds (compounds **2**, **6**, and **9**), showed a significant increase in quantity (*p* < 0.05) and five of the remaining eight (compounds **1**, **3**, **5**, **8**, and **11**) showed a decrease, with compounds **3** and **5** having a significant decrease (*p* < 0.05). The decrease induced by gamma radiation in the levels of these compounds may be attributed to the possible formation of irradiation-induced degradation products and/or free radicals [15]. The same decreasing effect in the compounds happened in a previous study by Pereira et al. [27], where the effects of gamma radiation on the phenolic profile of the infusions of *Thymus vulgaris* L. were analyzed. Several studies were performed and the results diverge according to several factors, such as the plant species studied, type of irradiation, and applied doses [28,29].

It should be highlighted that, at up to 400 μg/mL, none of the irradiated samples showed hepatotoxicity, evaluated in PLP2 cells, contrarily to the toxicity observed for the positive control ellipticine (concentration responsible for 50% of inhibition of the net cell growth—GI_50_ = 3.22 ± 0.67 μg/mL). These results are in agreement with previous results of irradiated samples of *T. vulgaris* and *Mentha x piperita* L. [30].

The calibration parameters of instrumentation (linear range, correlation coefficient (*R*^2^), equations of linear regression, limits of detection (LOD) and limits of quantification (LOQ) for AFB1 and OTA are shown in Table 3. The analytical methods for quantification of the two mycotoxins in samples of dried aromatic plants were further validated. Table 4 displays the accuracy and precision of the OTA and AFB1 analysis methods Recovery, as well as repeatability relative standard deviation (RSD_r_), and reproducibility relative standard deviation (RDS_R_), are within recommended ranges [31].

Data presented in Table 5 show the effect of gamma radiation doses (1, 5, and 10 kGy) on the reduction of AFB1 and OTA in dried leaves of *A. citrodora*. Assays were carried out in powdered samples spiked with 30 ng/g of AFB1 and OTA. This concentration was selected because it is an average value commonly used in this type of studies. When compared with non-irradiated samples (0 kGy), rates of mycotoxin reduction at different irradiation doses (1, 5, and 10 kGy) ranged between 21.2 and 22.6 ng/g for OTA, and 19.8 to 21,9 ng/g for AFB1, with no statistically significant differences (*p* > 0.05) between irradiated and non-irradiated samples, independently of the applied dose. No apparent dose-dependent effect was detected on the rate of mycotoxins decrease, either. These results suggest that irradiation at the tested doses, including the maximum allowed dose of 10 kGy, is not an effective treatment for AFB1 and OTA decontamination of dried plants.

The effect of gamma radiation on mycotoxin decontamination has been investigated in several food products (spices, feedstuff, coffee beans, fruits, seeds, vegetables, cured meat, and others), but divergent results have been reported. Some studies report high effectiveness of gamma radiation on the reduction of mycotoxin levels in various low moisture foods [32,33,34,35,36], although in some cases this effect is only observed at irradiation doses of 30 to 60 kGy [34,35], higher than the allowed dose of 10 kGy. In general, for the admissible dose by EU regulations, most reports conclude that no significant positive effects on mycotoxin decontamination are obtained for low moisture content foods or feeds [34,35,37,38,39,40,41]. In a study performed by Jalili et al. [35], gamma radiation was applied to black and white pepper and they found significant AF and OTA reductions only at irradiation doses of 30 kGy or higher and, even at 60 kGy, gamma rays were not completely effective in destroying those mycotoxins. At 10 kGy, mycotoxin reduction varied between 1.4% in OTA to 7.2% in AFB1 for samples with 12% of moisture content.

The reduced effect of this technique in low moisture matrices seems to be a direct result of the reduced water content. The presence of water is an important factor in the destruction of AF and OTA by gamma radiation, since water radiolysis leads to the formation of highly reactive free radicals that degrade the mycotoxins [42,43]. This effect has been demonstrated in a study by Kumar et al. [40], where the elimination of OTA in coffee grains with different moisture contents (9%, 10%, 12%, and 23%) was tested. OTA degradation in the lowest moisture content grains was 5% at 10 kGy, similar to the one obtained in our study, and 90% for the highest moisture content samples. In the present study, mycotoxin degradation by gamma radiation has been tested in herbs after drying, at the stage of ready-to-use product. To avoid the limited effect of the treatment generally observed for low moisture products (namely dried herbs), future studies on mycotoxin detoxification of herbs by gamma radiation should contemplate the fresh product, before drying. As previously stated, the treatment of high moisture matrices (fresh herbs) should result in higher detoxification effects. As concluded by several studies (e.g., [44,45,46]), the application of irradiation treatments to herbs while, in the fresh stage, should not negatively influence their nutritional value.

## 3. Materials and Methods

### 3.1. Safety Considerations

All recommended security considerations were taken into account when handling AF and OTA, due to the toxicity of these substances [47]. Protective equipment was used when handling solutions and all materials were decontaminated by autoclaving before disposal. The reusable materials were decontaminated during 12 h, immersed in a bleach solution of 10%, then immersed in acetone solution of 5%, during 1 h and finally washed with distilled water several times.

### 3.2. Samples and Sample Preparation

Dry leaves of *A. citrodora* (1000 g), whose common name is lemon verbena, and belonging to the family Verbenaceae, were provided by a local producer (Pragmático Aroma Lda, Alfândega da Fé, Bragança, Portugal). Water activity (aw) of the leaves was measured using a Rotronic HygroPalm AW1 equipment (Rotronic Instruments Ltd., Crawley, West Sussex, UK) and revealed to be 0.51. Leaves were then reduced to a fine powder with the aid of a kitchen blender, fully homogenized, and divided in two sets, one for irradiation tests and the other for in-house method validation. The material was preserved in sealed bags at 20 °C until further use.

### 3.3. Spiking with Mycotoxins

To test the effect of irradiation on mycotoxin reduction, dried powdered material was spiked with 30 ng/g of AFB1 and OTA, carefully homogenized, divided into 5 g aliquots and packaged in appropriate bags (polyethylene, 63 μm thickness). Aliquots were submitted to three different irradiation treatments: 1, 5, and 10 kGy. Each irradiation dose was applied to three aliquots in two independent treatments, for a total of six replicates for each dose. Non-irradiated samples (*n* = 6) were used as control (0 kGy).

### 3.4. Irradiation Treatment

Irradiation was performed in a ^60^Co experimental chamber (Precisa 22, Graviner Manufacturing Company Ltd., London, UK), following a procedure previously described by Pereira et al. [21]. The estimated dose rate was 1.7 kGy/h and the absorbed gamma radiation doses were 1.2 ± 0.1 kGy, 5.2 ± 0.2 kGy and 10.4 ± 0.4 kGy. The dose uniformity ratios (D_max_/D_min_) was 1.2. In order to simplify the values, 0 was considered for non-irradiated sample and 1, 5 and 10 kGy were considered irradiated samples. Samples were stored at −18 °C until further analysis were performed.

### 3.5. Phenolic Compounds Analysis

Samples (1 g) were extracted by maceration with 25 mL of methanol/H_2_O (80:20) during 1 h at 25 °C and 150 rpm following the procedure described by Pereira et al. [48].

The extracts were analyzed using an HPLC-DAD (Agilent Technologies, Santa Clara, CA, USA), connected to a mass spectrometer (MS) equipped with an ESI source and a hybrid triple quadrupole/linear ion trap mass analyzer (API 3200 Qtrap, Applied Biosystems, Darmstadt, Germany) [49]. The phenolic compounds were identified by comparing their retention times, UV-VIS and mass spectra with those obtained with standard compounds, when available. Otherwise, compounds were tentatively identified comparing the obtained information with available data reported in the literature. For quantitative analysis, a calibration curve for each available phenolic standard was constructed based on the UV signal. For the identified phenolic compounds for which a commercial standard was not available, the quantification was performed through the calibration curve of another compound from the same phenolic group. Results were expressed as mg per gram of extract.

### 3.6. Cytotoxicity Evaluation in Porcine Liver Cells

Cytotoxicity of the extracts was evaluated in porcine liver cells (PLP2) using the SRB assay, previously described by Abreu et al. [50]. The extracts described above (Section 3.5) were re-dissolved in water to a final concentration of 8 mg/mL. Ellipticine was used as a positive control.

### 3.7. Mycotoxin Analysis

#### 3.7.1. Aflatoxin Extraction and Quantification

For AFB1 extraction, half of the irradiated samples (2.5 g) were extracted by maceration (25 °C at 150 rpm) with sodium chloride (0.5 g) and methanol/water (20 mL, 80:20, *v*/*v*) for 30 min. The mixture was then filtered by gravity through a Whatman No. 4 filter paper (Sigma-Aldrich Co., St. Louis, MO, USA) and an aliquot (10 mL) of the filtrate was diluted with a portion of water (40 mL). The extract was homogenized and further filtered through a Whatman glass microfiber filter (934-AH). Subsequently, the filtered extract (20 mL) was purified through immunoaffinity column (AflaTest WB, VICAM, Watertown, MA, USA) by gravity, at a rate of approximately 1–2 drops/s. The column was washed a first time with phosphate-buffered saline with Tween (PBS-T: NaCl (8 g), Na_2_HPO_4_ (12 g), KH_2_PO_4_ (0.2 g), KCl (0.2 g), Tween 20 (0,1 mL) made up to 1000 mL with deionized water and the pH value was adjusted to 7.0 with NaOH), followed by a second wash with ultra-pure water (1 mL). AFB1 was eluted with 2 mL of methanol, collected in a glass vial, filtered through 0.2 µm nylon filters (Whatman) and analyzed by HPLC with fluorescence detection (FLD).

Samples were analyzed using a HPLC system (Smartline, Knauer, Berlin, Germany) coupled to a photochemical post-column derivatization reactor (PHRED unit, Aura Industries, New York, NY, USA), a fluorescence detector (FP-2020, Jasco, Easton, MD, USA) set to λ_ex_ 365 nm and λ_em_ 435 nm and using the Clarity 2.4 Software (DataApex, Prague, Czech Republic). The compounds were separated using an isocratic elution with a reverse-phase C18 column (100 mm × 4.6 mm, Merck Chromolith Performance, Darmstadt, Germany) at 35 °C (7971 R Grace oven). The mobile phase consisted of a mixture with acetonitrile/methanol/water (10:30:60, *v*/*v*/*v*) with a flow rate of 1 mL/min and the injection volume was 0.01 mL. AFB1 was identified by chromatographic comparison with the standard (Aflatoxin B1, Biopure, Tulln, Austria) and quantification was based on the fluorescence signal response.

#### 3.7.2. Ochratoxin A Extraction and Determination

OTA extraction followed the procedure described by Zhao et al. [51] with some modifications. Briefly, the other half of the irradiated samples (2.5 g) were extracted by stirring (25 °C at 150 rpm) with MeOH/1% NaHCO_3_ solution (12.5 mL, 70:30, *v*/*v*) for 30 min and subsequently filtered through Whatman No. 4 filter paper. Afterwards, the extract (10 mL) was diluted with PBS-T (40 mL) and further filtered through a Whatman glass microfiber filter (934-AH). The filtered extract (20 mL) was purified through an Ochratest WB immunoaffinity column (VICAM, Watertown, MA, USA) and the column was washed first with PBS-T (10 mL) and then with ultra-pure water (10 mL). Afterwards OTA was eluted with methanol (2 mL), collected in a glass vial, filtered through 0.2 µm nylon filters (Whatman) and analysed by HPLC-FLD.

OTA samples were analysed using the HPLC system and column described above for AF analysis, but without the derivatization process. The fluorescence detector was set to λ_ex_ 330 nm and λem 463 nm, mobile phase consisted of a mixture with acetonitrile/water/acetic acid (70:29.5:0.5, *v*/*v*/*v*), with a flow rate of 0.8 mL/min, and the injection volume was 10 μL. OTA was identified by chromatographic comparison with the standard (OTA standard solutions, Sigma Aldrich Co.) and quantification was based on the fluorescence signal response.

#### 3.7.3. In-House Method Validation

Stock solutions of AFB1 (5 μg/mL) and OTA (1 mg/mL) were prepared and stored at −20 °C. Working standard solutions of each mycotoxin (100 ng/mL) were prepared from stock solutions daily. Precision and recovery were performed by spiking the blank sample with two different mycotoxin concentrations: 10 ng/g and 30 ng/g of AFB1 and OTA, and one set of unspiked sample was used as a blank. Each sample set was composed of six replicates and tested in two different days (three replicates each day).

Instrumentation calibration parameters were determined following the methodology previously described by Arita et al. [52] and the recovery rates were determined from the six replicates of the two spiking levels, by calculation of the ratio of recovered AFB1 and OTA concentration relative to the known spiked concentration. Precision was calculated in terms of intraday repeatability (*n* = 3) and intermediate precision (interday within laboratory reproducibility; two different days) for each mycotoxin, at the two contamination levels in spiked samples.

Linearity, limit of detection (LOD), and limit of quantification (LOQ) were determined by three series of analyses, using 11 standard solutions with concentrations ranging from 0.05 ng/mL to 20 ng/mL of AFB1 and OTA. LOD and LOQ were calculated according to the following equations [52]: LOD = 3 × (*sa/b*) and LOQ = 10 × (*sa/b*), where *sa* is the standard deviation of the intercept of the regression line obtained from the calibration curve, and *b* is the slope of the line.

### 3.8. Statistical Analysis

Data analysis was performed using a one-way analysis of variance (ANOVA) followed by Tukey’s HSD test (*p* = 0.05) using a SPSS v. 23.0 software (IBM Corp., Armonk, NY, USA).

## 4. Conclusions

Gamma radiation was tested as a preservation and decontamination technique in dried leaves of *A. citrodora*. Regarding phenolic composition, doses 1 and 10 kGy showed some differences in the results, being that, at the lowest dose (1 kGy), three of the phenolic compounds (compounds **2**, **6**, and **9**) out of the eleven identified compounds, showed a significant increase in quantity (*p* < 0.05). Five of the remaining eight (compounds **1**, **3**, **5**, **8**, and **11**) showed a decrease, with compounds **3** and **5** having significant decrease (*p* < 0.05). On the other hand, at the highest dose (10 kGy), only compound **3** significantly increased, while compounds **2**, **4**, **5**, **6**, and **9** decreased, significantly. The same trend follows through with the TCP, TPA, TF, and TPC. Therefore, irradiation demonstrated a slight decrease in some of the identified phenolic compounds.

At the assayed doses, the irradiation process, did not induce detectable hepatotoxicity. Treatments at 1, 5, and 10 kGy doses were not effective in significantly decreasing OTA and AFB1. Thus, the legislated maximum dose of 10 kGy is ineffective to decrease decontamination using the studied mycotoxins.

In the case where mycotoxin decontamination of herbs is the primary goal of irradiation, and considering water content as an important parameter in the destruction of mycotoxins by gamma rays, future research should contemplate testing the effectiveness of gamma radiation in herbs prior to drying. For this matter, other features such as physical and chemical characteristics should also be studied.

## Figures and Tables

**Table 1:** Retention time (R*_t_*), wavelengths of maximum absorption in the visible region (λ_max_), mass spectral data (MS and MS^2^) and tentative identification of phenolic compounds in *Aloysia citrodora*.

Peak	R*_t_* (min)	λ_max_ (nm)	Molecular Ion [M − H]^−^ (*m*/*z*)	MS^2^ (*m*/*z*)	Tentative Identification	References
1	4.5	280	461	315 (8), 135 (28)	Verbasoside	[23,24]
2	15.1	344	637	351 (100), 285 (89)	Luteolin-7-*O*-diglucuronide	[22,23,24]
3	16.8	314	163	119 (100)	*p*-Coumaric acid	-
4	17.7	338	621	351 (100), 269 (20)	Apigenin-7-*O*-diglucuronide	[22]
5	18.2	330	623	461 (18), 315 (5)	Verbascoside	[22,23,24]
6	20.3	350	651	351 (100), 299 (5)	Chrysoeriol-7-*O*-diglucuronide	[23,24]
7	20.6	330	623	461 (18), 315 (5)	Isoverbascoside	[22]
8	21.3	330	623	461 (15), 315 (10)	Forsythoside	[23]
9	21.8	350	491	315 (100), 300 (23)	Isorhamnetin-3-*O*-glucuronide	-
10	23.2	330	637	491 (5), 461 (60), 315 (13)	Eukovoside	[22,23,24]
11	29.2	330	651	505 (7), 475 (22)	Martinoside	[23,24]

**Table 2:** Tentative identification of phenolic compounds (mg/g extract) in *A. citrodora* submitted to irradiation treatments at 1, 5, and 10 kGy, compared with non-irradiated samples (0 kGy).

Peak	Phenolic Compounds	0 kGy	1 kGy	5 kGy	10 kGy
1	Verbasoside ^1^	0.118 ± 0.001a	0.110 ± 0.01a	0.125 ± 0.02a	0.140 ± 0.03a
2	Luteolin-7-*O*-diglucuronide ^2^	18.9 ± 0.08b	19.1 ± 0.02a	18.6 ± 0.05c	18.1 ± 0.8d
3	*p*-Coumaric acid ^3^	1.14 ± 0.01b	1.07 ± 0.03c	1.13 ± 0.03b	1.20 ± 0.04a
4	Apigenin-7-*O*-diglucuronide ^4^	1.79 ± 0.03ab	1.81 ± 0.04a	1.71 ± 0.04b	1.61 ± 0.05c
5	Verbascoside ^1^	71.6 ± 0.24a	69 ± 0.95b	69 ± 0.71b	69.5 ± 0.47b
6	Chrysoeriol-7-*O*-diglucuronide ^4^	2.93 ± 0.01c	3.27 ± 0.05a	3.04 ± 0.04b	2.80 ± 0.04d
7	Isoverbascoside ^1^	0.74 ± 0.03a	0.79 ± 0.04a	0.73 ± 0.04a	0.67 ± 0.02a
8	Forsythoside ^1^	1.67 ± 0.03a	1.65 ± 0.20a	1.71 ± 0.15a	1.76 ± 0.10a
9	Isorhamnetin-3-*O*-glucuronide ^5^	1.63 ± 0.03b	1.75 ± 0.06a	1.51 ± 0.05c	1.27 ± 0.04d
10	Eukovoside ^1^	1.00 ± 0.03a	1.00 ± 0.04a	1.05 ± 0.06a	1.11 ± 0.09a
11	Martinoside ^1^	0.57 ± 0.01a	0.56 ± 0.04a	0.62 ± 0.08a	0.67 ± 0.11a
	TCP	75.8 ± 0.2a	73 ± 1b	73 ± 1b	73.8 ± 0.2b
	TPA	1.14 ± 0.01b	1.07 ± 0.03c	1.13 ± 0.03b	1.20 ± 0.04a
	TF	25.22 ± 0.03b	25.96 ± 0.09a	24.9 ± 0.1c	23.7 ± 0.1d
	TPC	102.1 ± 0.2a	100 ± 1b	99.3 ± 0.6b	98.8 ± 0.2b

The results are presented as the mean ± SD; (*n* = 12). TCP—total caffeoyl phenylethanoid derivatives (including verbasoside); TPA—total hydroxycinnamic acids; TF—total flavonoids; TPC—total phenolic compounds. Calibration curves: ^1^ caffeic acid (*y* = 359*x* + 488.4; *R*^2^ = 0.997); ^2^ Luteolin-7-*O*-glucoside (*y* = 334.2*x* − 261.39; *R*^2^ = 0.999); ^3^ *p*-coumaric acid (*y* = 706.09*x* + 1228.1; *R*^2^ = 0.9994); ^4^ apigenina-7-*O*-glucoside (*y* = 214.33*x* − 165.38; *R*^2^ = 0.999); ^5^ isorhametin-3-*O*-rutinoside (*y* = 284.12*x* + 67.055; *R*^2^ = 0.999). In each row different letters mean significant differences (*p* < 0.05).

**Table 3:** Calibration parameters of instrumentation for aflatoxin B1 and ochratoxin A detection and quantification.

Standard		AFB_1_	OTA
R*_t_* (retention time)	Min	6.79	2.20
	CV, % (*n* = 11)	0.76	2.45
Calibration curve		*y* = 312.36*x* − 27.24	*y* = 362.40*x* − 31.13
Correlation coefficient (*R*^2^)		0.999	0.999
Linearity range (ng/mL)		20 to 0.05	20 to 0.05
Limits	LOD ^a^ (ng/mL)	0.6	0.5
	LOQ ^b^ (ng/mL)	1.9	1.7

*R*^2^: Correlation coefficient; CV: coefficient of variation; ^a^ LOD: limit of detection of the chromatographic method; ^b^ LOQ: limit of quantification of the chromatographic method.

**Table 4:** Accuracy and precision of the analytical methods for aflatoxin B_1_ and ochratoxin A for spiking levels of 10 ng/g and 30 ng/g.

	AFB_1_		OTA	
	10 ng/g	30 ng/g	10 ng/g	30 ng/g
Mean Recovery (%)	88.3	88.9	76.4	92.0
RSD_r_ (%) ^a^	8.3–14.4	0.1	2.5–9.3	5.1
RSD_R_ (%) ^b^	3.3	-	5.6	-
Recommended Range (European Regulation No. 401/2006)				
Recovery (%)	70–110			
RSD_r_ (%)	<21	<22	<21	<22
RSD_R_ (%)	<32	<34	<32	<34

^a^ RSD_r_: Repeatability relative standard deviation; ^b^ RSD_R_: Reproducibility relative standard deviation.

**Table 5:** Reduction (ng/g; mean ± SD; *n* = 6) of aflatoxin B1 and ochratoxin A in spiked dried samples (30 ng/g of each mycotoxin) of *Aloysia citrodora* submitted to irradiation treatments at 1, 5, and 10 kGy, in comparison with non-irradiated samples (0 kGy).

Irradiation Dose	Mycotoxin Decrease (ng/g)	
	AFB_1_	OTA
0 kGy	21.9 ± 3.5 ^a^	22.6 ± 0.8 ^a^
1 kGy	20.7 ± 0.4 ^a^	21.5 ± 1.0 ^a^
5 kGy	19.8 ± 1.2 ^a^	21.2 ± 1.5 ^a^
10 kGy	20.4 ± 1.4 ^a^	21.4 ± 0.7 ^a^

^a^ No significant differences (*p* < 0.05) between any of the results were observed.

## References

[1] Lubbe A., Verpoortea R. (2011). Cultivation of medicinal and aromatic plants for specialty industrial materials. Ind. Crops Prod..

[2] Skotti E., Anastasaki E., Kanellou G., Polissiou M., Tarantilis P.A. (2014). Total phenolic content, antioxidant activity and toxicity of aqueous extracts from selected Greek medicinal and aromatic plants. Ind. Crops Prod..

[3] Acosta-Estrada B.A., Gutiérrez-Uribe J.A., Serna-Saldívar S.O. (2014). Bound phenolics in foods, a review. Food Chem..

[4] Wan Ainiza W.M., Jinap S., Sanny M. (2015). Simultaneous determination of aflatoxins and ochratoxin A in single and mixed spices. Food Control.

[5] The International Agency for Research on Cancer (IARC) (2002). Some Traditional Herbal Medicines, Some Mycotoxins, Naphthalene and Styrene.

[6] Rodrigues P., Venâncio A., Lima N. (2012). Mycobiota and mycotoxins of almonds and chestnuts with special reference to aflatoxins. Food Res. Int..

[7] Lee D., Lyu J., Lee K.-G. (2015). Analysis of aflatoxins in herbal medicine and health functional foods. Food Control.

[8] Romagnoli B., Menna V., Gruppioni N., Bergamini C. (2007). Aflatoxins in spices, aromatic herbs, herb-teas and medicinal plants marketed in Italy. Food Control.

[9] Harris J.P., Mantle P.G. (2001). Biosynthesis of ochratoxins by *Aspergillus ochraceus*. Phytochemistry.

[10] Majeed S., Iqbal M., Asi M.R., Iqbal S.Z. (2013). Aflatoxins and ochratoxin A contamination in rice, corn and corn products from Punjab, Pakistan. J. Cereal Sci..

[11] Waśkiewicz A., Beszterda M., Bocianowski J., Golińnski P. (2013). Natural occurrence of fumonisins and ochratoxin A in some herbs and spices commercialized in Poland analyzed by UPLCeMS/MS method. Food Microbiol..

[12] Ashiq S., Hussain M., Ahmad B. (2014). Natural occurrence of mycotoxins in medicinal plants: A review. Fungal Genet. Biol..

[13] Santos L., Marín S., Sanchis V., Ramos A.J. (2009). Screening of mycotoxin multi contamination in medicinal and aromatic herbs sampled in Spain. J. Sci. Food Agric..

[14] Mizani M., Sheikh N., Ebrahimi S.N., Gerami A., Tavakoli F.A. (2009). Effect of gamma irradiation on physico-mechanical properties of spice packaging films. Radiat. Phys Chem..

[15] Alothman M., Bhat R., Karim A.A. (2009). Effects of radiation processing on phytochemicals and antioxidants in plant produce. Trends Food Sci. Technol..

[16] Byun M.W., Yook H.S., Kim K.S., Chung C.K. (1999). Effects of gamma irradiation on physiological effectiveness of Korean medicinal herbs. Radiat. Phys. Chem..

[17] Sádecká J. (2007). Irradiation of spices—A review. Czech J. Food Sci..

[18] Nagy T.O., Solar S., Sontag G., Koenig J. (2011). Identification of phenolic components in dried spices and influence of irradiation. Food Chem..

[19] Farkas J., Mohácsi-Farkas C. (2011). History and future of food irradiation. Trends Food Sci. Technol..

[20] (1999). Directive 1999/3/EC of the European Parliament and of the Council of 22 February 1999 on the establishment of a Community list of foods and food ingredients treated with ionising radiation. Off. J. Eur. Communities.

[21] Pereira E., Antonio A.L., Barreira J.C.M., Barros L., Bento A., Ferreira I.C.F.R. (2015). Gamma irradiation as a practical alternative to preserve the chemical and bioactive wholesomeness of widely used aromatic plants. Food Res. Int..

[22] Bilia A.R., Giomi M., Innocenti M., Gallori S., Vincieri F.F. (2008). HPLC–DAD–ESI–MS analysis of the constituents of aqueous preparations of verbena and lemon verbena and evaluation of the antioxidant activity. J. Pharm. Biomed. Anal..

[23] Quirantes-Piné R., Funes L., Micol V., Segura-Carretero A., Fernández-Gutiérrez A. (2009). High-performance liquid chromatography with diode array detection coupled to electrospray time-of-flight and ion-trap tandem mass spectrometry to identify phenolic compounds from a lemon verbena extract. J. Chromatogr. A.

[24] Quirantes-Piné R., Arráez-Román D., Segura-Carretero A., Fernández-Gutiérrez A. (2010). Characterization of phenolic and other polar compounds in a lemon verbena extract by capillary electrophoresis-electrospray ionization-mass spectrometry. J. Sep. Sci..

[25] Alipieva K., Korkina L., Orhan I.E., Georgiev M.I. (2014). Verbascoside—A review of its occurrence, (bio)synthesis and pharmacological significance. Biotechnol. Adv..

[26] Gonçalves S., Grevenstuk T., Martins N., Romano A. (2015). Antioxidant activity and verbascoside content in extracts from two uninvestigated endemic *Plantago* spp.. Ind. Crops Prod..

[27] Pereira E., Barros L., Antonio A.L., Cabo Verde S., Santos-Buelga C., Ferreira I.C.F.R. (2016). Infusion from *Thymus vulgaris* L. treated at different gamma radiation doses: Effects on antioxidant activity and phenolic composition. LWT Food Sci. Technol..

[28] Ramabulana T., Mavunda R.D., Steenkamp P.A., Piater L.A., Dubery I.A., Madala N.E. (2015). Secondary metabolite perturbations in *Phaseolus vulgaris* leaves due to gamma radiation. Plant Physiol. Biochem..

[29] Ramabulana T., Mavunda R.D., Steenkamp P.A., Piater L.A., Dubery I.A., Madala N.E. (2016). Perturbation of pharmacologically relevant polyphenolic compounds in Moringa oleifera against photo-oxidative damages imposed by gamma radiation. J. Photochem. Photobiol. B.

[30] Pereira E., Pimenta A.I., Calhelha R.C., Antonio A.L., Cabo Verde S., Barros L., Santos-Buelga C., Ferreira I.C.F.R. (2016). Effects of gamma irradiation on cytotoxicity and phenolic compounds of *Thymus vulgaris* L. and *Mentha x piperita* L.. LWT Food Sci. Technol..

[31] European Union (2006). Commission Regulation (EC) No 401/2006 of 23 February 2006 laying down the methods of sampling and analysis for the official control of the levels of mycotoxins in foodstuffs. Off. J. Eur. Communities.

[32] Aziz N.H., Moussa L.A.A. (2004). Reduction of fungi and mycotoxins formation in seeds by gamma-radiation. J. Food Saf..

[33] Iqbal S.Z., Bhatti I.A., Asi M.R., Zuber M., Shahid M., Parveen I. (2003). Effect of γ-irradiation on fungal load and aflatoxins reduction in red chillies. Radiat. Phys. Chem..

[34] Jalili M., Jinap S., Noranizan A. (2010). Effect of gamma radiation on reduction of mycotoxins in black pepper. Food Control.

[35] Jalili M., Jinap S., Noranizan M.A. (2012). Aflatoxins and ochratoxin a reduction in black and white pepper by gamma radiation. Radiat. Phys. Chem..

[36] Prado G., de Carvalho E.P., Oliveira M.S., Madeira J.G.C., Morais V.D., Correa R.F., Cardoso V.N., Soares T.V., da Silva J.F.M., Goncalves R.C.P. (2003). Effect of gamma irradiation on the inactivation of aflatoxin B1 and fungal flora in peanut. Braz. J. Microbiol..

[37] Akueche E.C., Anjorin S.T., Harcourt B.I., Kana D., Adeboye E., Shehu I., Akande R., Adeleke A.T., Shonowo O.A., Adesanmi C.A. (2012). Studies on fungal load, total aflatoxins and ochratoxin A contents of gamma-irradiated and non-irradiated *Sesamum indicum* grains from Abuja markets, Nigeria. Kasetsart J. Nat. Sci..

[38] Herzallah S., Alshawabkeh K., Al Fataftah A. (2008). Aflatoxin decontamination of artificially contaminated feeds by sunlight, gamma-radiation, and microwave heating. J. Appl. Poult Res..

[39] Hooshmand H., Kloperstein C.F. (1995). Effects of gamma irradiation on mycotoxin disappearance and amino acid contents of corn, wheat, and soybeans with different moisture contents. Plant Foods Hum. Nutr..

[40] Kumar S., Kunwar A., Gautam S., Sharma A. (2012). Inactivation of *A. ochraceus* spores and detoxification of ochratoxin A in coffee beans by gamma irradiation. J. Food Sci..

[41] Vita D.S., Rosa P., Giuseppe A. (2014). Effect of Gamma Irradiation on Aflatoxins and Ochratoxin A Reduction in Almond Samples. J. Food Res..

[42] Calado T., Venâncio A., Abrunhosa L. (2014). Irradiation for mold and mycotoxin control: A Review. Compr. Rev. Food Sci. Food Saf..

[43] Rustom I.Y.S. (1997). Aflatoxin in food and feed: Occurrence, legislation and inactivation by physical methods. Food Chem..

[44] Fan X., Sokorai K.J.B. (2008). Retention of quality and nutritional value of 13 fresh-cut vegetables treated with low-dose radiation. J. Food Sci..

[45] Koike A., Barreira J.C.M., Barros L., Santos-Buelga C., Villavicencio A.L.C.H., Ferreira I.C.F.R. (2015). Irradiation as a novel approach to improve quality of *Tropaeolum majus* L. flowers: Benefits in phenolic profiles and antioxidant activity. Innov. Food Sci. Emerg. Technol..

[46] Rezende A.C.B., Igarashi M.C., Destro M.T., Franco B.D.G.M., Landgraf M. (2014). Effect of Gamma Radiation on the Reduction of *Salmonella* strains, *Listeria monocytogenes*, and Shiga Toxin-Producing *Escherichia coli* and Sensory Evaluation of Minimally Processed Spinach (*Tetragonia expansa*). J. Food Prot..

[47] Castegnaro M., Hunt S.C., Sansone E.B., Schuller P.L., Siriwardana B.G., Telling G.M., van Egmond H.P., Walker E.A. (1980). Laboratory decontamination and destruction of aflatoxins B1, B2, G1 and G2 in laboratory wastes. IARC Publications No. 37.

[48] Pereira E., Barros L., Dueñas M., Antonio A.L., Santos-Buelga C., Ferreira I.C.F.R. (2015). Gamma irradiation improves the extractability of phenolic compounds in *Ginkgo biloba* L.. Ind. Crops Prod..

[49] Barros L., Pereira E., Calhelha R.C., Dueñas M., Carvalho A.M., Santos-Buelga C., Ferreira I.C.F.R. (2013). Bioactivity and chemical characterization in hydrophilic and lipophilic compounds of *Chenopodium ambrosioides* L.. J. Funct. Foods.

[50] Abreu R.M.V., Ferreira I.C.F.R., Calhelha R.C., Lima R.T., Vasconcelos M.H., Adega F., Chaves R., Queiroz M.J.R.P. (2011). Anti-hepatocellular carcinoma activity using human HepG2 cells and hepatotoxicity of 6-substituted methyl 3-aminothieno[3,2-*b*]pyridine-2-carboxylate derivatives: In vitro evaluation, cell cycle analysis and QSAR studies. Eur. J. Med. Chem..

[51] Zhao X., Yuan Y., Zhang X., Yue T. (2014). Identification of ochratoxin A in Chinese spices using HPLC fluorescent detectors with immunoaffinity column cleanup. Food Control.

[52] Arita C., Calado T., Venâncio A., Lima N., Rodrigues P. (2014). Description of a strain from an atypical population of *Aspergillus parasiticus* that produces aflatoxins B only, and the impact of temperature on fungal growth and mycotoxin production. Eur. J. Plant Pathol..

